# A Simple and Effective Method to Enhance the Mechanical Properties, Dimensional Stability, and Mildew Resistance of Bamboo Scrimber

**DOI:** 10.3390/polym15204162

**Published:** 2023-10-20

**Authors:** Jiayu Xu, Zhezhe Zhou, Xiaochun Zhang, Yantao Xu

**Affiliations:** 1 College of Chemistry and Materials Engineering, Zhejiang A & F University, Hangzhou 311300, China; 18767300889@163.com; 2 Zhejiang Provincial Collaborative Innovation Center for Bamboo Resources and High-Efficiency Utilization, Hangzhou 311300, China; 3 Beijing Key Laboratory of Wood Science and Engineering, Beijing Forestry University, Beijing 100107, China; 15968102826@163.com

**Keywords:** bamboo scrimber, alkali treatment, anti-mildew, mechanical properties, dimensional stability

## Abstract

Given the increasingly prominent contradiction between the supply of and demand for wood, the abundant resource of bamboo can be a good substitute. Bamboo scrimber can effectively improve the utilization rate of bamboo and has good mechanical properties. However, bamboo scrimber has the problem of poor mildew resistance, and does not meet the requirements for outdoor applications. In this study, in order to further improve the mildew resistance and mechanical properties of bamboo scrimber, alkali treatment was used to remove some nutrients from the bamboo bundles and change the pH of the bamboo scrimber. The results showed that nutrients such as hemicellulose, lignin, starch, and sugar were notably removed from bamboo bundles, and the pH of bamboo was slightly alkaline. The anti-mildew effect was significantly enhanced, which could allow use in outdoor environments, and the mechanical properties and dimensional stability were also improved. Among them, TB6 bamboo scrimber showed comprehensively excellent properties. The infection time in the laboratory mildew test increased from 3 days to more than 30 days, and the infection time in the outdoor mildew resistance test increased from 1 week to more than 8 weeks; the static bending intensity of TB6 increased by 62.6% to 150 MPa, and the bending modulus increased by 71.7% to 14.2 GPa; the change rate of water absorption thickness was reduced to 0.58%. This modification method effectively improved the mildew resistance of bamboo scrimber, while maintaining high mechanical strength, and provides a new method for the outdoor application of bamboo scrimber.

## 1. Introduction

With the rapid development of the economy, the demand for wood in various fields is increasing, and the problem of wood shortage is becoming more prominent [1,2]. In addition, in recent years, some countries have taken a series of measures to limit the logging of timber as a means of timber protection. Bamboo is an ideal building material, with the advantages of high yield, short growth period, high strength, and good toughness [3,4]. Therefore, the utilization of bamboo is of great significance in relieving the pressure on wood resources. With the rapid development of the bamboo processing industry, a large number of bamboo composite materials, especially bamboo scrimber, laminated bamboo and plybamboo have been developed [5]. Compared with other bamboo composite materials, bamboo scrimber contains more raw materials, including small-sized bamboo, so the utilization rate of bamboo scrimber is higher [6,7]. However, bamboo contains a large amount of nutrients such as starch and sugar, making bamboo-based materials susceptible to erosion by mold and rotting fungi, resulting in a decrease in density and strength, discoloration of the material surface, and a serious impact on its service life [8]. Therefore, it is necessary to develop bamboo scrimber with excellent mechanical strength and mildew resistance [9].

In order to extend the service life of bamboo, many methods have been adopted, including physical and chemical modifications. Physical modification methods include high-temperature carbonization [10], ultrasonic processing [11], heat treatment [12,13], and microwave treatment [14]. Physical mildew prevention methods are simple processes with a low cost, but they cannot guarantee that bamboo will not be corroded by mold over a long time. Chemical modification methods include soaking, acetylation [15,16], and resin modification [17,18], etc. Chemical mildew control methods are effective and durable, but they are costly and complex to operate, and can also cause environmental pollution and harm human health. Furthermore, many microbes have developed resistance to used chemicals [19]. Therefore, it is necessary to develop a new and effective method to improve the mildew resistance of bamboo.

**Figure 1:**
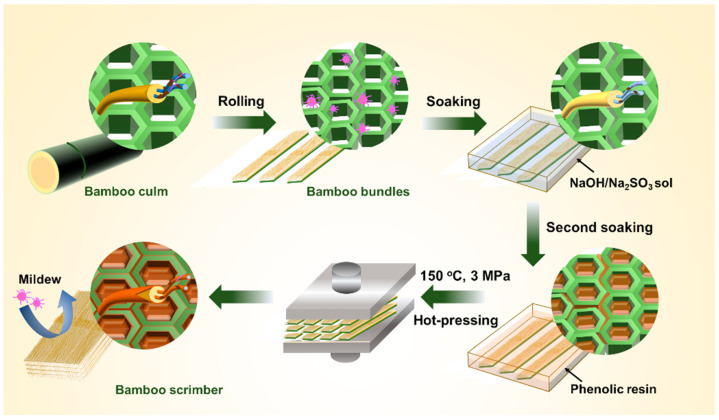
Schematic diagram of the preparation process and internal structure change of mildew resistant bamboo scrimber.

In this work, we developed bamboo scrimber with mildew resistance and good mechanical properties (Figure 1). First, a sodium hydroxide (NaOH) and sodium sulfite (Na_2_SO_3_) impregnating process was used to remove most of the hemicellulose, lignin, starch, sugars, and other nutrients; at the same time, the internal pH of bamboo was increased, which helped to improve its mildew resistance. Then, the bamboo bundles were impregnated and hot-pressed to form a dense structure, resulting in bamboo scrimber with excellent mechanical properties.

## 2. Experimental Section

### 2.1. Materials

Bamboo bundles (1000 mm × 20 mm × 5 mm) and phenolic resin were purchased from Zhejiang Yongyu Furniture Co., Ltd. (Taizhou, China). Bamboo bundles were cut in 150 mm × 20 mm × 5 mm. NaOH and Na_2_SO_3_ were purchased from Nanjing Chemical Reagent Co., Ltd. (Nanjing, China). All chemicals used in the experiments were of analytical reagent grade. *Aspergillus niger*, *Penicillium citrinum*, and *Trichoderma viride* were received from the lab.

### 2.2. Treatment of Bamboo Bundles

First, 2.5 mol·L^−1^ NaOH and 0.4 mol·L^−1^ Na_2_SO_3_ solutions were prepared. The bamboo bundles were impregnated in a mixture of 80 °C for 0, 0.5, 1, 2, 4, 6, 8 and 10 h, respectively. After treatment, the bamboo bundles were rinsed with deionized water and dried until the moisture content reached 10%.

### 2.3. Preparation of Bamboo Scrimer

The treated bamboo bundles were soaked in phenolic resin at room temperature for 15 min, then pressed at 150 °C and 3 MPa for 18 min. The size of the bamboo scrimber was 150 mm × 150 mm × 15 mm, and the average density was 1.0 g·cm^−1^. The bamboo bundles and bamboo scrimber prepared by alkali treatment 0.5, 1, 2, 4, 6, 8, and 10 h were named TB0.5, TB1, TB2, TB4, TB6, TB8, and TB10, respectively, and the control group was named NB.

### 2.4. Anti-Mildew Property

The anti-mildew property was evaluated according to the Chinese national standard GB/T 18261 [20]. In this study, *Aspergillus niger*, *Penicillium citrinum* and *Trichoderma viride* were used for mildew resistance testing. Bamboo scrimber and mold were incubated at 28 °C and 85% relative humidity for 30 days. Outdoor mildew tests were carried out during the warm and humid season. The samples were stacked in the natural environment, exposing them to the natural conditions of mold infection. The test ended when the surface of the control sample was completely infected with mold. The prevention efficiency was evaluated based on Table 1 and Equation (1).

(1) $$E=\left(1-\frac{D_{1}}{D_{0}}\right)\times 100$$

where D_1_ is the average infection value (AIV) of the treated sample and D_0_ is the AIV of control sample.

### 2.5. Mechanical Properties

Flexural strength and modulus of elasticity were tested according to Chinese national standard GB/T 17657-2013 using a universal testing machine [21]. Three-point bending test was performed on 5 samples in each group. The size of sample was 150 mm× 10 mm× 5 mm.

### 2.6. Dimensional Stability

Dimensional stability was tested according to GB/T 17657. The specimens were placed in a constant temperature and humidity chamber with a temperature of 23 ± 2 °C and relative humidity of (50 ± 3)% for at least 72 h for balanced treatment. Part of the specimens were placed in an electric thermostatic drying oven with a temperature of 70 ± 2 °C for 24 h and the length of the samples was measured. The remaining specimens were placed in the constant temperature and humidity chamber with a temperature of 40 ± 2 °C and relative humidity of 90–95%. After treatment for 96 ± 1 h, their length and thickness were measured to obtain the total size change rate. The length change rate and thickness change rate were calculated according to Equations (2) and (3).

(2) $$∆L=\frac{l_{2}-l_{1}}{l_{1}}\times 100$$

(3) $$∆t=\frac{t_{2}-t_{1}}{t_{1}}\times 100$$

where l_1_ represents the initial length of the sample, l_2_ represents the final length of the sample, t_1_ represents the initial thickness of the sample, and t_2_ represents the final thickness of the sample.

### 2.7. pH Measurement

The pH of bamboo scrimber was measured according to the Chinese national standard GB/T 6043-2009 [22]. Three grams of bamboo powder was accurately weighed, placed in a 50 mL beaker; 30 mL of distilled water, free of carbon dioxide, was accurately measured, mixed for 5 min, and then stirred for 15 min. After standing for 20 min, pH value was measured.

### 2.8. Chemical Analysis

The samples were sliced along the transverse direction, mounted on a specimen holder with a conductive adhesive, then gilded with sputtering. Scanning electron microscopy (SEM, S2020641400 TM3030, Hitachi, Tokyo, Japan) was used to observe the cross-section morphology of alkali treated samples and untreated samples under the accelerated voltage of 15 kV. The bamboo bundles were characterized by Fourier transform infrared spectrometry (FTIR, S20023024 IR Prestige-21, Shimadzu, Kyoto, Japan). Each group of samples was ground into powder and pressed with KBr, and the wavenumber was between 4000 and 400 cm^−1^. The crystal structure changes of the sample fibers were detected by X-ray diffraction (XRD, LabX XRD-6000, Shimadzu, Kyoto, Japan), and all samples were pulverized into powder using a small, low-speed grinder. XRD used Cu Kα (λ = 1.5418 nm) radiation, 2θ range of 5–60°, and step size of 0.02°. The crystallinity (CrI) of cellulose before and after treatment was calculated by the method of Segal [23]:

(4) $$CrI=\left(\frac{I_{200}-I_{am}}{I_{200}}\right)\times 100\%$$

where CrI is the relative crystallinity, I_200_ is the diffraction peak of 200 crystal plane, and I_am_ is the diffraction intensity of amorphous region.

The relative contents of cellulose, hemicellulose, and lignin in the bamboo bundles were calculated by Van Soest method [24]. The content of bamboo starch was determined by UV spectrophotometry, and 1.0 g of dried bamboo powder was weighed and placed into a beaker, and 70 mL of distilled water was added for boiling. After boiling, the starch solution was immediately pumped and filtered to obtain bamboo starch filtrate. The absorbance value of bamboo starch solution at the peak absorption wavelength of 580 nm was determined using an ultraviolet spectrophotometer. The soluble sugar content of bamboo was determined using a UV spectrophotometer: 1.5 g of sample powder was weighed and 30 mL distilled water was added and heated at 50 °C for 30 min. The absorbance value was measured at 490 nm.

## 3. Results and Discussion

### 3.1. Chemical Characterization

**Figure 2:**
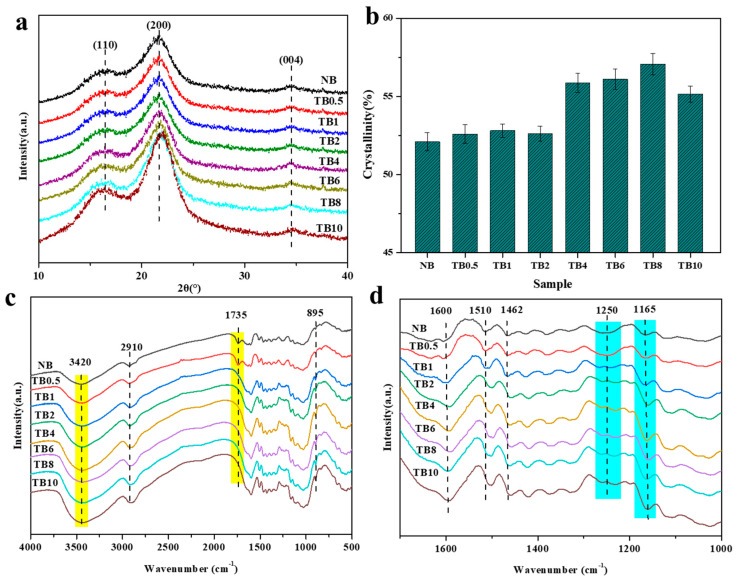
XRD and FTIR of control sample and treated sampled. (**a**) XRD of control sample and treated sampled. (**b**) Crystallinity of control sample and treated sampled. (**c**) FTIR spectrum of 4000–500 cm^−1^. (**d**) FTIR spectra of 1700–1000 cm^−1^.

Figure 2a shows the XRD pattern of the sample before and after alkali treatment. The results showed that the location of the diffraction peak of cellulose in bamboo did not change much, indicating that alkali treatment has little effect on the crystal area of cellulose, and the crystal structure of bamboo cellulose has not been damaged [25]. The diffraction peaks of all bamboo bundles were 16.6°, 21.9°, and 34.6°, representing the (110), (200), and (004) planes of cellulose, respectively. As the alkali treatment time prolonged, the intensity of the I_200_ crystal plane diffraction peaks gradually increased. The relative crystallinity of each sample was calculated according to the Segal method and Figure S1. Among them, the peak intensity of I_am_ (18.3°) and I_200_ was used to calculate the crystallinity [26]. The calculation results of crystallinity are shown in Figure 2b. The crystallinity of NB was 52.1%, and as the alkali treatment time was prolonged, the crystallinity of the sample gradually increased, with TB8 having the highest crystallinity (57.1%), 10.8% higher than NB. This is because the removal of hemicellulose and the extraction of pectin exposed the hydroxyl groups of amorphous microfibers, forming hydrogen bonds with the microfibers on the surface of the crystallization zone, thereby improving the crystallinity of bamboo fibers [27]. However, the crystallinity of TB10 slightly decreased, mainly due to the dissolution of lignin and hemicellulose, as well as the reaction between the alkali and the crystallization zone, which caused the destruction of the crystal structure, resulting in a slight decrease in crystallinity.

FTIR was used to analyze the effect of alkali treatment on the chemical composition of bamboo, as shown in Figure 2c,d. The absorption peaks at 895 cm^−1^, 1165 cm^−1^, 2910 cm^−1^, and 3420 cm^−1^ belong to the C–H stretching vibration peak, C–O–C stretching vibration peak, –CH_2_ stretching vibration peak and O–H absorption peak in the molecular structures of cellulose, respectively [28,29,30]. As shown in Figure S2a,d, after alkali treatment, the characteristic peak intensity at 3420 cm^−1^ and 1165 cm^−1^ increased, indicating that alkali treatment increased cellulose relative content. In the control group, there was an absorption peak at 1735 cm^−1^, which disappeared after 1 h of alkali treatment (Figure S2b). This peak was the stretching vibration absorption peak of non-conjugated C=O of hemicellulose [31,32]. The disappearance of this peak indicated that the hemicellulose had been destroyed, which may have been due to the hydrolysis reaction between hemicellulose and alkali. The absorption peaks at 1600 cm^−1^, 1510 cm^−1^, 1462 cm^−1^, and 1250 cm^−1^ belong to the aromatic skeletal vibration, asymmetric C–H bending vibration from –OCH_3_, and C–O stretching of the syringyl ring and ester in the molecular structures of lignin [31,32,33]. The intensity of 1250 cm^−1^ decreased after alkali treatment (Figure S2c), indicating that alkali treatment also reduced lignin content. These results are consistent with the analysis of XRD above.

### 3.2. Composition and Morphology Characterization

**Figure 3:**
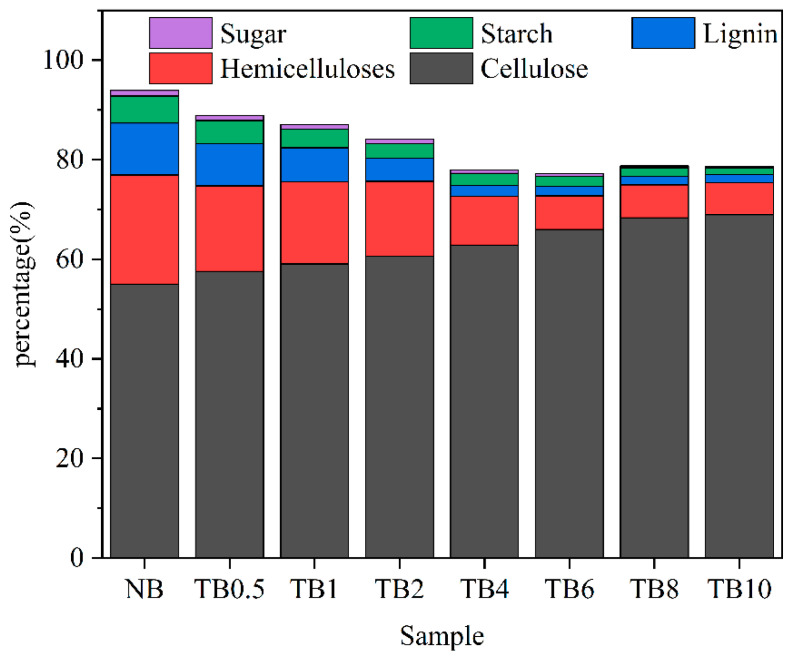
Chemical composition untreated bamboo and treated bamboo.

As shown in Figure 3, the chemical components of bamboo after different alkali treatment times were quantitatively determined by the Van Soest method. The relative contents of cellulose, hemicellulose and lignin in control sample were 55 wt%, 21.9 wt% and 10.4 wt% respectively. The relative contents of lignin and hemicellulose decreased with the increase of alkali treatment time. When the alkali treatment time was 6 h, the relative content of lignin decreased to 1.9 wt%, while at 8 h and 10 h, the relative content decreased to 0.05 wt%. The hemicellulose and lignin were removed in the modified bamboo bundles, and the relative content of cellulose increased; this was consistent with FTIR and XRD analysis. The starch and sugar in bamboo are the main factors that contribute to its susceptibility to mold. As shown in Figure 3, with the increase in treatment time, the content of starch and sugar in alkali-treated bamboo significantly decreased. In untreated bamboo, the starch content and sugar content were 5.44% and 1.13%, respectively. After 6 h of treatment, the starch content and sugar content of modified bamboo decreased to 1.99% and 0.57%, respectively. This is mainly because a portion of amylose and sugar are dissolved in water. In addition, alkaline solution caused amylose to gelatinize and further dissolve in water, directly reducing the content of starch and sugar in bamboo. The nutrients in the modified bamboo scrimber were reduced, which helped to improve its mildew resistance.

**Figure 4:**
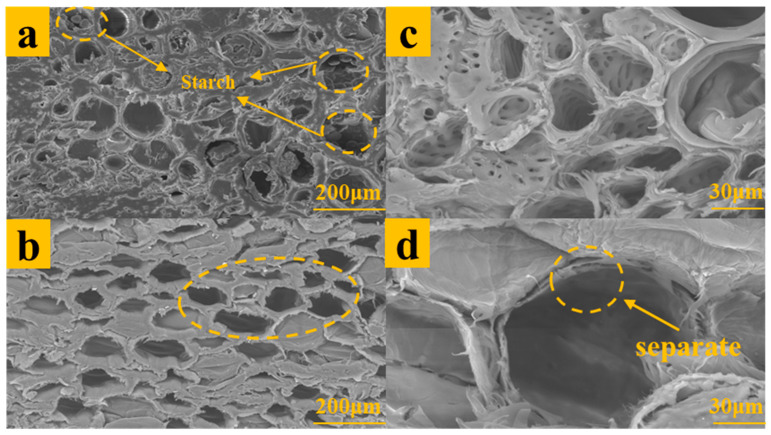
The cross-sectional microstructure of the sample. (**a**,**c**) The cross-sectional micromorphology of NB. (**b**,**d**) The cross-sectional micromorphology of TB6.

The parenchyma cells of the untreated bamboo scrimber were intact (Figure 4c), while the treated parenchyma cells collapsed and separated (Figure 4d). This is also because after alkali treatment, part of the lignin and hemicellulose in bamboo were removed, and a large number of hydrogen bonds were formed between cellulose, leading to the collapse and stratification of the parenchyma cells. The untreated parenchyma cells of bamboo scrimber contained a lot of starch particles (Figure 4a), while these substances disappeared in the parenchyma cells treated with alkali (Figure 4b), which was consistent with the results of previous studies [34,35]; this result was also consistent with that of the starch content above. This is because after alkali treatment, the starch was gelatinized and dissolved in the solution, and part of parenchyma cells broke, so that gelatinized starch particles can more easily precipitate from the cell. Alkali treatment degraded nutrients such as starch in bamboo, thus improving the mildew resistance of bamboo. These findings are the same as the results of chemical composition characterization.

### 3.3. Anti-Mildew Property

**Figure 5:**
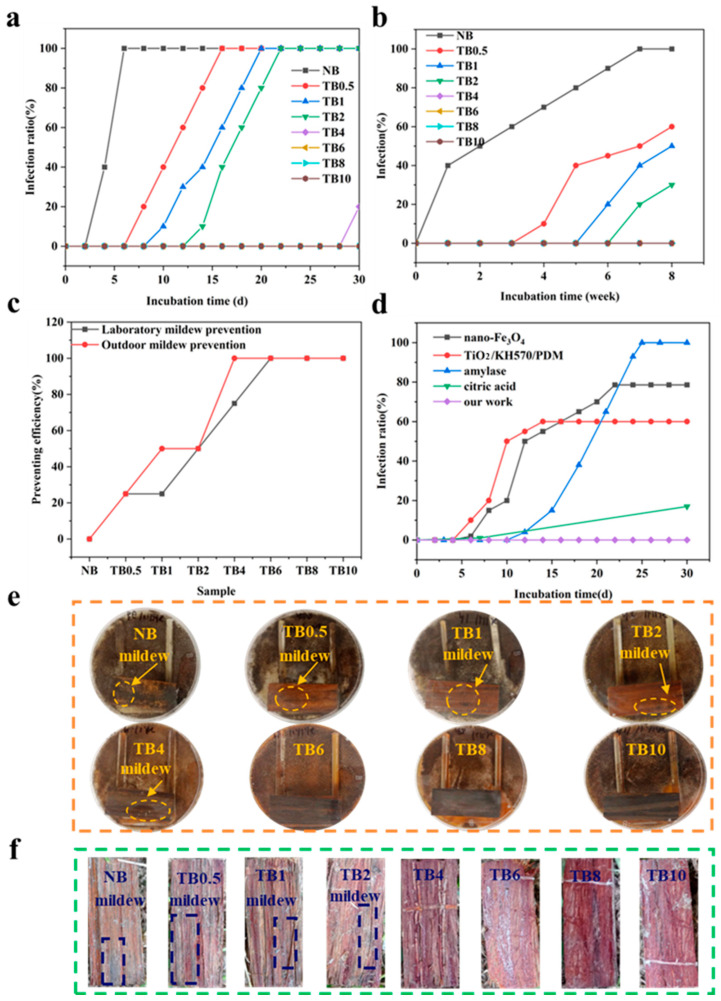
Laboratory mildew test and outdoor mildew test. (**a**) Laboratory mildew test infection. (**b**) Outdoor mildew test infection. (**c**) The efficacy of laboratory and outdoor mildew prevention tests. (**d**) Comparison of our work with other different processes for preparing mildew-resistant bamboo. (**e**) Image of bamboo scrimber infected with mold after 30 days in the laboratory mildew test. (**f**) Image of bamboo scrimber infected with mold after 8 weeks in the outdoor mildew test.

*Aspergillus niger*, *Penicillium citrinum*, and *Trichoderma viride* were selected for mildew resistance testing, and the mixed bacteria were incubated with the samples for 30 days (Figure 5a). Obviously, the control sample had poor mildew resistance, as the infection rate was 100% during over an incubation period of only 6 days. After alkali treatment, the infection rate of mold decreased and the mildew resistance increased. After 8 days of incubation with the mixed sample, the TB0.5 sample first developed mold. On the 10th day, TB1 sample also contracted mold. On day 14, the infection rate of TB1 reached 40%, and mold infection began to appear in the sample of TB2. On day 22, the entire surface of TB2 was infected. Until 30 days of incubation, the TB6 sample was not infected, and the control efficiency of these samples reached 100% (Figure 5a,e). Outdoor mildew testing was carried out during the warm and humid season. NB started to mold in the first week, and the surface was covered with mold in 7 weeks (Figure 5b). Three weeks later, TB0.5 was also infected, with a final infection of about 50%. TB1 and TB2 were moldy at weeks 5 and 6, respectively. By the end of the test, no mildew was found in the samples treated for 4 h or more (Figure 5b,f). Figure 5c shows the efficacy of laboratory and outdoor mildew prevention tests. TB6, TB8, and TB10 exhibited excellent anti-mold performance. This is because alkali treatment dissolved nutrients such as starch and sugar in water, which directly reduced the content of nutrients in bamboo, resulting in insufficient nutrition for the mold, thus inhibiting the growth and reproduction of mold. Figure 5d shows a comparison of the mildew resistance of bamboo obtained from our work with other different treatments [1,36,37,38]. As can be seen, our work achieved effective mildew prevention over a longer period of time.

The optimal pH value for common molds such as *Aspergillus niger*, *Penicillium viridis*, and *Aspergillus flavus* is 4–6 [39], while most bamboo is acidic, with pH ranging from 4.8 to 6.6, which is also one of the reasons for serious mildew of bamboo. The pH value of untreated bamboo was 7.06 (Table 2), the pH values of alkali-treated bamboo were greater than 7, and the pH value of TB10 reached 8. After alkali treatment, the pH value of bamboo deviates from the optimal pH range of mold growth, affecting the activity of mold metabolic enzymes and nutrient absorption, which is also one of the reasons for the decrease in mold growth on bamboo after alkali treatment.

### 3.4. Mechanical Properties

**Figure 6:**
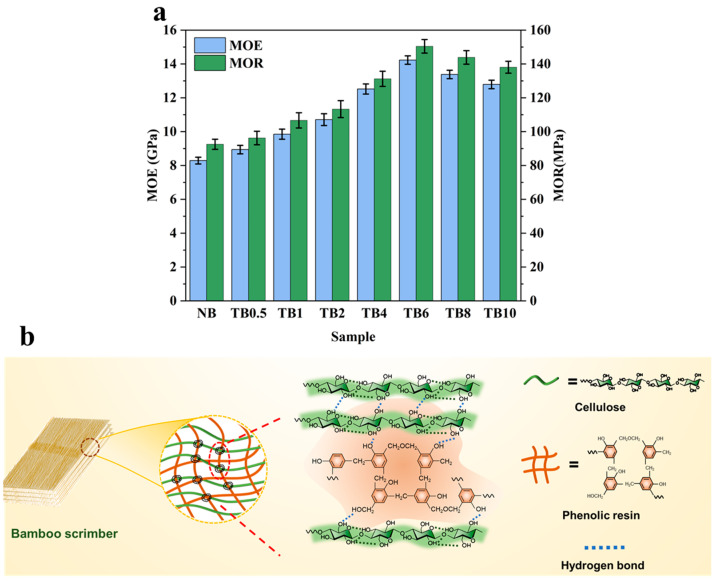
Mechanical properties of untreated and treated samples. (**a**) MOE and MOR of untreated and treated bamboo scrimber. (**b**) Diagram of cellulose and phenolic resin binding.

The mechanical properties of bamboo scrimber were measured by three-point bending test, as shown in Figure 6a. The flexural strength (MOE) and flexural modulus (MOR) of untreated bamboo scrimber were 8.29 GPa and 92.5 MPa, respectively. Compared with the untreated samples, the mechanical properties of the samples treated with alkaline were obviously improved. With the extension in alkali treatment time, the mechanical properties of bamboo scrimber first increased and then decreased. The MOR of TB6 was the highest, which was 62.6% higher than that of NB, reaching 150 MPa, and its MOE was also the highest, which was 71.7% higher than that of the NB, reaching 14.2 GPa. The improvement in mechanical properties of TB6 can be attributed to the removal of most of the hemicellulose and lignin in the bamboo bundle, exposing more cellulose surfaces. The large number of hydroxyl groups on the surface of cellulose interact to form hydrogen bonds, thereby increasing the density of hydrogen bonds [40]; this is consistent with FTIR and XRD analysis. In addition, after alkali treatment, bamboo cell walls of porosity increased, the intercellular layer is removed, basically forming micron-grade of directional bamboo cellulose fiber. Therefore, phenolic resin is more easily immersed in cell walls and intercellular spaces, generating hydrogen bonds and van der Waals forces with bamboo, thereby improving the mechanical properties of bamboo, as shown in Figure 6b. After alkali treatment for 8 h and 10 h, the mechanical properties of the modified bamboo scrimber decreased, but the MOE and MOR were still higher than those of the control. This is because the chemical treatment time is too long, and too much lignin and hemicellulose are removed, so the overall structure of bamboo cannot be maintained, resulting in a decrease in mechanical properties [41].

### 3.5. Dimensional Stability

In a hot and dry environment, the ambient humidity decreases and the length of sample reduces, because the water in the bamboo scrimber spreads into the surrounding environment, causing the sample to shrink, which results in a decrease in length. In high-humidity environments, the size of the sample increased with increasing environmental humidity. This is because external water enters bamboo fibers through pipelines, increasing the size of the fiber. The change rates of thickness and length of NB are relatively high, reaching 1.94% and 0.58%, respectively. This is because hemicellulose and cellulose in bamboo contain lots of hydrophilic free hydroxyl groups. Under the high-humidity environment, water fully enters the cell wall and cell cavity. The free hydroxyl groups on cellulose and hemicellulose adsorb water molecules from the environment through hydrogen bonds and molecular forces, leading to swelling, which ultimately leads to the increase in bamboo size [42]. The size change rate of the alkali-treated sample was lower than that of the untreated bamboo scrimber. The size change rate of TB2, TB4, and TB6 were lower, with the thickness change rate below 1% and the length change rate between 0.15% and 0.17% (Table 3), indicating that TB2, TB4, and TB6 have good dimensional stability. This is because in a highly alkaline solution, hemicellulose and lignin are degraded, and a large number of hydrophilic groups are removed, thereby reducing the hygroscopic property of bamboo scrimber. In contrast, during alkali treatment, the removal of lignin and hemicellulose results in increased cell wall porosity, which provides space for the penetration of phenolic resin. Therefore, the bamboo fibers are completely covered with phenolic resin, preventing the entry of water [43,44,45]. In summary, highly alkaline solution can degrade hemicellulose and lignin in bamboo, reduce the moisture absorption performance of bamboo scrimber, and improve its dimensional stability.

## 4. Conclusions

This study used a process of alkali treatment, phenolic resin impregnation, and hot-pressing to prepare modified bamboo scrimber, which showed enhanced mildew resistance and dimensional stability while maintaining its high mechanical properties. The results showed that compared with untreated bamboo scrimber, the MOR of TB6 samples increased by 62.6% to 150 MPa, and the MOE increased by 71.7% to 14.2 GPa; the infection time in the laboratory mildew test increased from 3 days to more than 30 days, and the infection time in the outdoor mildew resistance increased from 1 week to more than 8 weeks; and the change rate of water absorption thickness also decreased to 0.58%. This occurred because: (1) alkali treatment partially removed some nutrients from the bamboo bundle and increased the pH value of the bamboo, changing the internal environment, inhibiting the growth and reproduction of mold, and improving the mildew resistance of the bamboo scrimber; (2) the partial removal of hemicellulose and lignin improved the connections between cellulose, enhancing the hydrogen bond density and van der Waals forces between fibers. Phenolic resins also generated hydrogen bonds and van der Waals forces with bamboo bundles after high-pressure densification, which further improved the mechanical properties of bamboo scrimber. This modification method can effectively improve the mechanical properties of bamboo and extend its service life, which is of great significance for expanding the applications of bamboo in outdoor buildings.

## Figures and Tables

**Table 1:** Sample infection value classification [20].

Infection Value	Sample Infected Area
0	No mycelium or mold
1	Surface infected area is less than 1/4
2	Surface infected area is between 1/4 and 1/2
3	Surface infected area is between 1/2 and 3/4
4	Surface infected area is over 3/4

**Table 2:** pH of untreated bamboo and treated bamboo.

Sample	pH
NB	7.06
TB0.5	7.53
TB1	7.54
TB2	7.71
TB4	7.73
TB6	7.86
TB8	7.85
TB10	8.03

**Table 3:** The size change rate of the samples with the environmental.

Sample	Thickness Change Rate (%)	Length Change Rate (%)
NB	1.94 ± 0.08	0.58 ± 0.04
TB0.5	1.57 ± 0.05	0.58 ± 0.01
TB1	1.38 ± 0.06	0.22 ± 0.01
TB2	0.96 ± 0.04	0.15 ± 0.03
TB4	0.69 ± 0.02	0.16 ± 0.01
TB6	0.58 ± 0.02	0.17 ± 0.02
TB8	0.62 ± 0.03	0.49 ± 0.03
TB10	0.60 ± 0.03	0.33 ± 0.02

## Data Availability

The data presented in this study are available on request from the corresponding author.

## Supplementary Materials

The following supporting information can be downloaded at: https://www.mdpi.com/article/10.3390/polym15204162/s1. The specific calculation of the crystallinity of the sample is shown in the supplementary information.

## References

[1] Wang Q., Han H., Lou Z., Han X., Wang X., Li Y. (2022). Surface property enhancement of bamboo by inorganic materials coating with extended functional applications. Compos. Part A Appl. Sci. Manuf..

[2] Chen J., Guagliano M., Shi M., Jiang X., Zhou H. (2021). A comprehensive overview of bamboo scrimber and its new development in China. Eur. J. Wood Wood Prod..

[3] Fang C.-H., Jiang Z.-H., Sun Z.-J., Liu H.-R., Zhang X.-B., Zhang R., Fei B.-H. (2018). An overview on bamboo culm flattening. Constr. Build. Mater..

[4] Huang Z., Sun Y. (2021). Experimental study on the surface light and thermal properties of bamboo. J. Build. Eng..

[5] Nkeuwa W.N., Zhang J.L., Semple K.E., Chen M.L., Xia Y.L., Dai C. (2022). Bamboo-based composites: A review on fundamentals and processes of bamboo bonding. Compos. Part B Eng..

[6] Yu Y., Zhu R., Wu B., Hu Y., Yu W. (2015). Fabrication, material properties, and application of bamboo scrimber. Wood Sci. Technol..

[7] Huang Z., Künzel H., Krus M., Zhang W. (2022). Three-dimensional tests on hygric properties of laminated bamboo and bamboo scrimber. J. Build. Eng..

[8] Zhang J., Zhang B., Chen X., Mi B., Wei P., Fei B., Mu X. (2017). Antimicrobial bamboo materials functionalized with ZnO and graphene oxide nanocomposites. Materials.

[9] Li H., Yang Z.B., Yang F., Gu Z.C., Liu R., Yu L.L., Ma X.X., Fei B.H. (2018). Influence of Four Coatings on the Mold-Resistance and Combustion Performance of Decorative Bamboo Curtain. Wood Fiber Sci..

[10] Lin J.T., Liu H., Wang S.Q., Huang J.D., Zhang W.B. (2023). Preparation and Influence Mechanism of High-Efficiency Bamboo-Based Conductive Carbon Powder by One-Step Carbonization. J. Biobased Mater. Bioenergy.

[11] Guan M.J., Huang Z.W., Zhu D.Y. (2022). The effect of ultrasonic process on the shear strength and the microstructure of the bonding interface of laminated bamboo lumber. Eur. J. Wood Wood Prod..

[12] Huang Y.X., Meng F.D., Liu R., Yu Y.L., Yu W.J. (2019). Morphology and supramolecular structure characterization of cellulose isolated from heat-treated moso bamboo. Cellulose.

[13] Lin Q.Q., Huang Y.X., Yu W.J. (2020). An in-depth study of molecular and supramolecular structures of bamboo cellulose upon heat treatment. Carbohydr. Polym..

[14] Poonia P.K., Deepa S.R., Kumar M., Kumar A. (2021). Viability of wood decaying fungal mycelium after microwave radiation of bamboo culm. Maderas-Cienc. Tecnol..

[15] Chee S.S., Jawaid M., Sultan M., Alothman O.Y., Abdullah L.C. (2019). Thermomechanical and dynamic mechanical properties of bamboo/woven kenaf mat reinforced epoxy hybrid composites. Compos. Part B Eng..

[16] Liang H.S., Xing H., Qin M., Wu H.J. (2020). Bamboo-like short carbon fibers@Fe_3_O_4_@phenolic resin and honeycomb-like short carbon fibers@Fe_3_O_4_@FeO composites as high-performance electromagnetic wave absorbing materials. Compos. Part A Appl. Sci. Manuf..

[17] Jhu Y.S., Hung K.C., Xu J.W., Wu J.H. (2019). Effects of acetylation on the thermal decomposition kinetics of makino bamboo fibers. Wood Sci. Technol..

[18] Papadopoulos A.N., Bikiaris D.N., Mitropoulos A.C., Kyzas G.Z. (2019). Nanomaterials and Chemical Modifications for Enhanced Key Wood Properties: A Review. Nanomaterials.

[19] Li J.P., Wu Z.X., Bao Y.J., Chen Y.H., Huang C.J., Li N., He S., Chen Z. (2017). Wet chemical synthesis of ZnO nanocoating on the surface of bamboo timber with improved mould-resistance. J. Saudi Chem. Soc..

[20] (2013). Test Method for Anti-Mildew Agents in Controlling Wood Mould and Stain Fungi.

[21] (2013). Test Methods of Evaluating the Poperties of Wood-Based Panels and Surface Decorated Wood-Based Panels.

[22] (2009). Method for Determination of pH Value of Wood.

[23] Segal L., Creely J.J., Martin A.E., Conrad C.M. (1959). An Empirical Method for Estimating the Degree of Crystallinity of Native Cellulose Using the X-Ray Diffractometer. Text. Res. J..

[24] Bartos A., Anggono J., Farkas A.E., Kun D., Soetaredjo F.E., Móczó J., Antoni, Purwaningsih H., Pukánszky B. (2020). Alkali treatment of lignocellulosic fibers extracted from sugarcane bagasse: Composition, structure, properties. Polym. Test..

[25] Liu K., Du H.S., Zheng T., Liu H.Y., Zhang M., Zhang R., Li H., Xie H., Zhang X., Ma M. (2021). Recent advances in cellulose and its derivatives for oilfield applications. Carbohydr. Polym..

[26] Oprea M., Voicu S.I. (2020). Recent advances in composites based on cellulose derivatives for biomedical applications. Carbohydr. Polym..

[27] Lin J.Y., Yang Z.X., Hu X.X., Hong G.H., Zhang S.B., Song W. (2018). The Effect of Alkali Treatment on Properties of Dopamine Modification of Bamboo Fiber/Polylactic Acid Composites. Polymers.

[28] Lu M.T., He W., Li Z., Qiang H., Cao J.Z., Guo F., Wang R., Guo Z. (2020). Effect of Lignin Content on Properties of Flexible Transparent Poplar Veneer Fabricated by Impregnation with Epoxy Resin. Polymers.

[29] Yang L., Lou Z., Han X., Liu J., Wang Z., Zhang Y., Wu X., Yuan C., Li Y. (2020). Fabrication of a novel magnetic reconstituted bamboo with mildew resistance properties. Mater. Today Commun..

[30] Ma C.Y., Wang H.M., Wen J.L., Shi Q.T., Wang S.F., Yuan T.-Q., Sun R.-C. (2020). Structural elucidation of lignin macromolecule from abaca during alkaline hydrogen peroxide delignification. Int. J. Biol. Macromol..

[31] Rao F., Ji Y.H., Yang Y., Zhang Y.H., Li N., Yu W., Chen Y. (2021). Rapid Process Natural Bamboo into Outdoor Bamboo-Fiber-Reinforced Composite with High Surface Photostability. Forests.

[32] Feng N.R., Liang Y.X., Hu D.Y. (2020). Delignified bamboo as skeleton matrix for shape-stable phase change heat storage material with excellent reversible thermochromic response property. J. Energy Storage.

[33] Dong Y.M., Liu X.Y., Liu J.J., Yan Y.T., Liu X.R., Wang K., Li J. (2021). Evaluation of anti-mold, termite resistance and physical-mechanical properties of bamboo cross-linking modified by polycarboxylic acids. Constr. Build. Mater..

[34] Chen H., Wu J., Shi J., Zhang W., Wang H. (2021). Effect of alkali treatment on microstructure and thermal stability of parenchyma cell compared with bamboo fiber. Ind. Crops Prod..

[35] Felisberto M.H.F., Beraldo A.L., Costa M.S., Boas F.V., Franco C.M.L., Clerici M.T.P.S. (2019). Characterization of young bamboo culm starch from *Dendrocalamus asper*. Food Res. Int..

[36] Huang X.D., Hse C.Y., Shupe T.F. (2014). Study on the Mould-Resistant Properties of Moso Bamboo Treated with High Pressure and Amylase. Bioresources.

[37] Lou Z., Han X., Liu J., Ma Q., Yan H., Yuan C., Yang L., Han H., Weng F., Li Y. (2021). Nano-Fe_3_O_4_/bamboo bundles/phenolic resin oriented recombination ternary composite with enhanced multiple functions. Compos. Part B Eng..

[38] Yu Z.X., Zhang X.F., Zhang R., Yu Y., Sun F.B. (2022). Improving the Mould and Blue-Stain-Resistance of Bamboo through Acidic Hydrolysis. Polymers.

[39] Liu Y.D. (2012). Bamboo timber mildew and anti-mold technology. Trans. Tech. Publ..

[40] Bacigalupe A., Molinari F., Eisenberg P., Escobar M.M. (2020). Adhesive properties of urea-formaldehyde resins blended with soy protein concentrate. Adv. Compos. Hybrid Mater..

[41] Chen C.J., Li Z.H., Mi R.Y., Dai J.Q., Xie H., Pei Y., Li J., Qiao H., Tang H., Yang B. (2020). Rapid Processing of Whole Bamboo with Exposed, Aligned Nanofibrils toward a High-Performance Structural Material. ACS Nano.

[42] Zhang X., Li J., Yu Y., Wang H. (2018). Investigating the water vapor sorption behavior of bamboo with two sorption models. J. Mater. Sci..

[43] Frühwald E. (2007). Effect of high-temperature drying on properties of Norway spruce and larch. Holz Als Roh-Und Werkst..

[44] Ishikura Y., Abe K., Yano H. (2010). Bending properties and cell wall structure of alkali-treated wood. Cellulose.

[45] Xie J., Qi J., Hu T., De Hoop C.F., Hse C.Y., Shupe T.F. (2016). Effect of fabricated density and bamboo species on physical–mechanical properties of bamboo fiber bundle reinforced composites. J. Mater. Sci..

