# Supplementary material for: A Simple and Effective Method to Enhance the Mechanical Properties, Dimensional Stability, and Mildew Resistance of Bamboo Scrimber
Source: Polymers (Basel). 2023 Oct 20;15(20):4162. doi: 10.3390/polym15204162 (PMC10610577; doi:10.3390/polym15204162)
Supplement: Supplementary file 1 [file polymers-15-04162-s001.zip › polymers-2661663-supplementary.pdf]

Supplementary Material

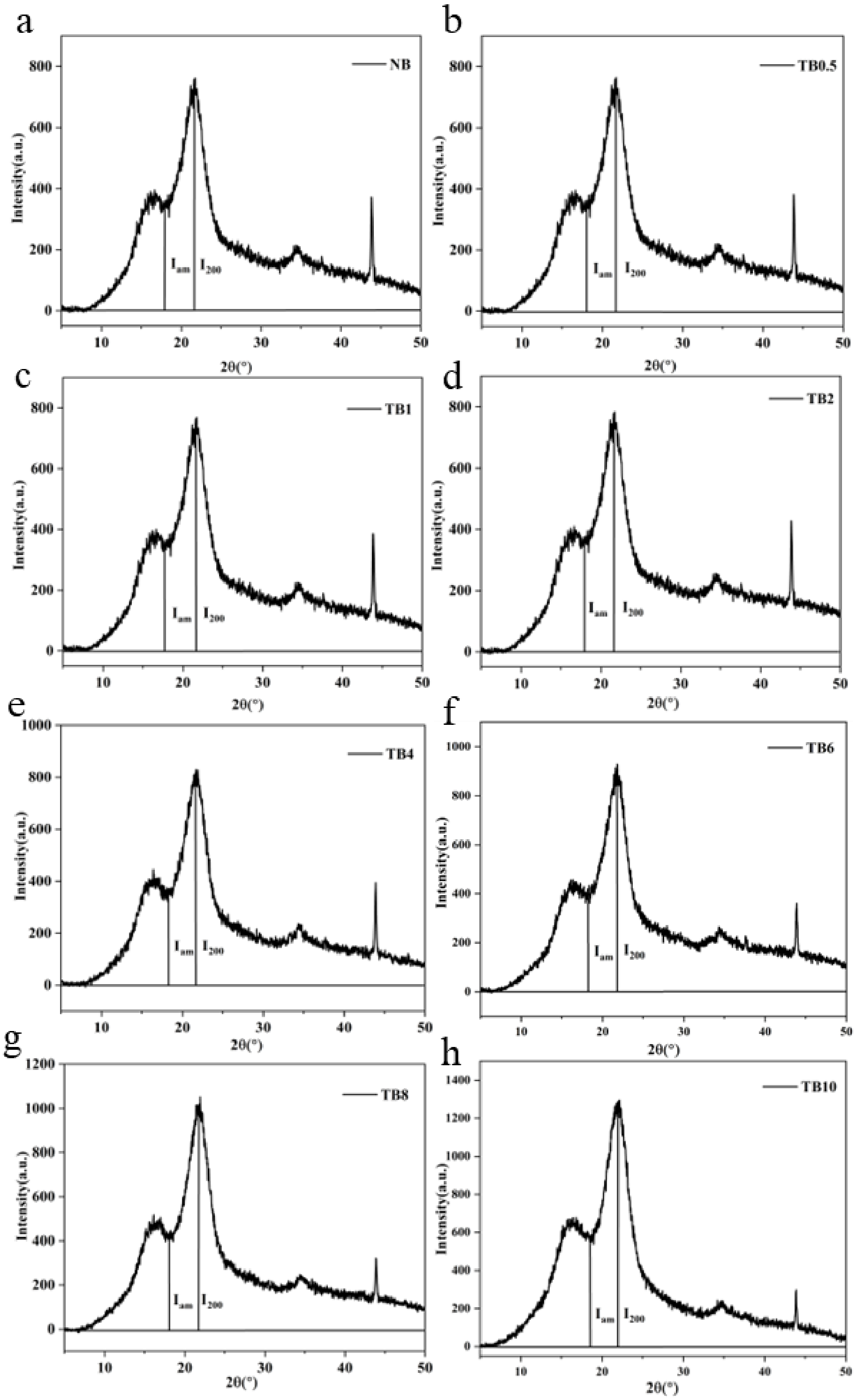

Fig. S1. XRD pattern of each sample. (a) XRD of NB. (b) XRD of TB0.5. (c) XRD of TB1. (d) XRD of TB2. (e) XRD of TB4. (f) XRD of TB6. (g) XRD of TB8. (h) XRD of TB10.

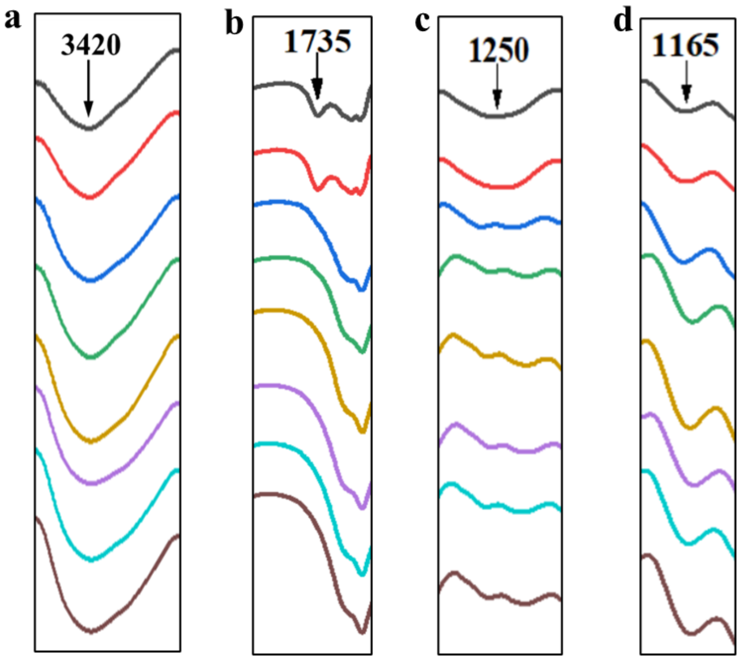

Fig. S2. Fourier transform infrared spectroscopy. (a): Enlarged image at 3420 cm^{-1}; (b): Enlarged image at 1735 cm^{-1}; (c): Enlarged image at 1250 cm^{-1}; (d): Enlarged image at 1165 cm^{-1}.
